# Supplementary material for: Safety Assessment and Probiotic Potential Comparison of Bifidobacterium longum subsp. infantis BLI-02, Lactobacillus plantarum LPL28, Lactobacillus acidophilus TYCA06, and Lactobacillus paracasei ET-66
Source: Nutrients. 2023 Dec 29;16(1):126. doi: 10.3390/nu16010126 (PMC10780348; doi:10.3390/nu16010126)
Supplement: Supplementary file 1 [file nutrients-16-00126-s001.zip › nutrients-2712551-supplementary.pdf]

Supplement data

1. Materials and Methods

Genomic Sequencing and Analysis

The whole-genome sequencing was executed utilizing PacBio third-generation sequencing technology. Comprehensive genomic maps were acquired for BLI-02, LPL28, and ET-66. Due to poor resolution, the genomic map of TYCA06 was assembled into four contigs. The genomic data were locally aligned to the downloaded Virulence Factor Database (VFDB) employing BLAST to detect potential virulence factors. The raw sequence data for *L. paracasei* ET-66 was further uploaded to the Comprehensive Antibiotic Resistance Database (CARD) for analysis, with the objective to identify potential antibiotic resistance genes. The identified genes were annotated based on their closest matches in the respective databases.

2. Results

2.1. VFDB analysis:

Upon local alignment with the Virulence Factor Database (VFDB setB), the analysis revealed the absence of pavA gene in BLI-02 and the presence of the pavA gene in three strains: LPL28, TYCA06, and ET-66. The pavA gene is originated from *Streptococcus pneumoniae*, and associated with bacterial adhesion to host cells in strains. Literature indicates the presence of a homologous gene in *L. casei* [1]. Additionally, there are references suggesting the relevance of this gene to the adhesion to human intestinal epithelial cells in *L. acidophilus* [2,3]. Given that the genus *Lactobacillus* is not pathogenic and the alignment similarity is only around 42%, this gene should not be regarded as a potential risk virulence factor.

2.2. CARD Analysis:

L. paracasei ET-66 displayed microbiological values exceeding the cut-off values for kanamycin and chloramphenicol established by the European Food Safety Authority (EFSA, 2012) and ISO (2010). A comprehensive genomic analysis, using the CARD database, was performed to identify potential antibiotic resistance genes in *L. paracasei* ET-66 (Table S1).

Table S1. *L. paracasei* ET-66 genes with ≥ 50% sequence similarity in CARD analysis.

Sequence similarity	Gene
70-80%	1. elfamycin-resistant EF-Tu<br>2. rifamycin-resistant beta-subunit of RNA polymerase (rpoB)<br>3. antibiotic-resistant fusA
60-70%	1. aminocoumarin-resistant gyrB
50-60%	1. antibiotic-resistant murA transferase<br>2. ATP-binding cassette (ABC) antibiotic efflux pump<br>3. major facilitator superfamily (MFS) antibiotic efflux pump<br>4. fluoroquinolone-resistant gyrA<br>5. fluoroquinolone-resistant gyrB<br>6. lsa-type ABC-F protein<br>7. streptogramin vat acetyltransferase<br>8. glycopeptide resistance gene cluster: vanR

	9. chloramphenicol acetyltransferase (CAT)
	10. daptomycin-resistant cls

3. Discussion

Although no discernible kanamycin resistance genes were identified with more than 50% similarity, a gene presenting a 50.98% similarity to chloramphenicol acetyltransferase was detected. Given its modest protein similarity of 50%, its attribution to significant resistance for chloramphenicol appears limited. The analysis also revealed genes on plasmids in ET-66. One potential gene, conferring resistance to salicylic acid antibiotic, showed a 20.05% identity. The top identity for the aligned genes did not exceed 30%, suggesting a reduced likelihood of equivalent antibiotic resistance and a low risk of resistance transmission. It's worth considering that these strains might inherently possess resistance mechanisms, potentially through multifunctional efflux pumps or the natural resistance due to a denser cell wall characteristic of Gram-positive bacteria.

References

1. Munoz-Provencio, D.; Perez-Martinez, G.; Monedero, V. Characterization of a fibronectin-binding protein from Lactobacillus casei BL23. *Journal of applied microbiology* **2010**, *108*, 1050-1059, doi:10.1111/j.1365-2672.2009.04508.x.
2. Hymes, J.P.; Johnson, B.R.; Barrangou, R.; Klaenhammer, T.R. Functional Analysis of an S-Layer-Associated Fibronectin-Binding Protein in Lactobacillus acidophilus NCFM. *Appl Environ Microbiol* **2016**, *82*, 2676-2685, doi:10.1128/AEM.00024-16.
3. Bisht, S.; Singh, K.S.; Choudhary, R.; Kumar, S.; Grover, S.; Mohanty, A.K.; Pande, V.; Kaushik, J.K. Expression of fibronectin-binding protein of L. acidophilus NCFM and in vitro refolding to adhesion capable native-like protein from inclusion bodies. *Protein Expr Purif* **2018**, *145*, 7-13, doi:10.1016/j.pep.2017.11.007.
